# The Cognitive Functions Dementia Battery: A Novel Computerized Tool for Neurocognitive Disorders

**DOI:** 10.14336/AD.2025.0412

**Published:** 2026-05-18

**Authors:** Raquel Lemos, Sofia Areias-Marques, Alexandros Lazaridis, Johanna Zils, Cristina Martins, Sílvia Almeida, Luísa Alves, David Brieber, Albino J. Oliveira-Maia

**Affiliations:** ^1^Champalimaud Research & Clinical Centre, Champalimaud Foundation, Lisboa, Portugal.; ^2^ISPA – Instituto Universitário de Ciências Psicológicas, Sociais e da Vida, Lisboa, Portugal.; ^3^SCHUHFRIED GmbH, Mödling, Austria.; ^4^CELGA-ILTEC, Faculdade de Letras da Universidade de Coimbra, Coimbra, Portugal.; ^5^Graduate Programme in Clinical and Health Psychology, Faculdade de Psicologia da Universidade de Lisboa, Lisboa, Portugal.; ^6^Hospital de Egas Moniz, Unidade Local de Saúde de Lisboa Ocidental, Lisboa, Portugal.; ^7^Centro Clínico Académico de Lisboa NOVA Medical School, Faculdade de Ciências Médicas, NMS, FCM, Universidade NOVA de Lisboa, Lisboa, Portugal.; ^8^NOVA Medical School, Faculdade de Ciências Médicas, NMS, FCM, Universidade NOVA de Lisboa, Lisboa, Portugal.

**Keywords:** Computerized battery, Cognitive Functions Dementia, validity, reliability, neurocognitive disorders

## Abstract

The Cognitive Functions Dementia (CFD) is a computerized battery to assess the cognitive domains included in the Diagnostic and Statistical Manual of Mental Disorders (DSM-5) criteria for neurocognitive disorders. We aimed at examining the psychometric properties and the clinical validity of the CFD for neurocognitive disorders. Psychometric characterization was conducted in healthy individuals, stratified according to age, sex, and education to represent a norming sample. Analyses included structural validity and measurement invariance, assessed through confirmatory factor analyses (CFA) and multi-group CFA, as well as internal consistency, and re-test reliability of the battery index values. Patients with major or minor neurocognitive disorder contributed data to compute receiver operating characteristics to assess diagnostic accuracy of the CFD indices relative to healthy participants. Data from 422 healthy volunteers and 81 patients were collected for the study. The confirmatory factor analysis model confirmed the validity of the structure proposed for CFD, with the five cognitive domains (attention, verbal long-term memory, expressive language, executive functions, and perceptual motor functions) describing an overall factor (CFD-Index). Good to excellent values of internal consistency and of re-test reliability were obtained for all indices. Importantly, CFD indexes were accurate in discriminating patients with neurocognitive disorders from healthy participants. The CFD battery is a valid and reliable computerized instrument to characterize patients with neurocognitive dysfunction.

## INTRODUCTION

Neuropsychological assessment is required in clinical and research settings to identify cognitive impairment in neurodegenerative or other neuropsychiatric disorders, namely in dementia, to monitor the progression of such impairments, as well as to plan interventions for these disorders and/or determine their effect [1]. The *Diagnostic and Statistical Manual of Mental Disorders* (DSM-5) [2] has replaced the term dementia by neurocognitive disorder (NCD), either major or mild, depending on the level of cognitive decline, and has defined that deterioration may be in a single cognitive domain, requiring standardized neuropsychological tests to objectively confirm the cognitive impairment [2]. More recently, similar innovations were adopted by the *International Statistical Classification of Diseases and Related Health Problems* (ICD-11) [3]. Thus, in the context of dementia, neuropsychological assessment is an essential component of the diagnostic process.

Traditionally, paper-and-pencil cognitive measures have been used in neuropsychological assessment, including screening instruments or comprehensive neuropsychological batteries. Cognitive screening instruments, such as the Mini-Mental State Examination (MMSE) [4] or the Montreal Cognitive Assessment (MoCA) [5], are widely used for early identification of moderate to severe cognitive impairment, since they are brief, easily applied as part of a routine clinical visit, can be used by clinicians without specific training, and provide a total score interpretable to monitor changes over time [6]. Nevertheless, limitations to brief cognitive screening instruments are also recognized, including poor accuracy in detecting milder forms of impairment and limitations in the use of adjusted scores for demographic variables known to influence cognitive performance, namely age and education [6, 7]. Comprehensive neuropsychological assessments, on the other hand, in addition to overcoming these limitations, provide detailed cognitive profiles of impaired and preserved functions, thus aiding in the identification of primary and secondary diagnoses, in determining the nature and severity of cognitive and functional difficulties, and in planning interventions such as cognitive rehabilitation [6]. The importance of comprehensive neuropsychological test batteries over cognitive screening tests was highlighted by the National Academy of Neuropsychology (NAN) in two position papers [6, 7].

Since the 1980s, assessments have started to be digitized in light of technological advances, the accessibility of portable computers, and the advent of the internet [8], with the creation of reliable and efficient instruments. This process has included the adaptation of pencil-and-paper tests to computer-administered versions, but also the development of new computerized tests [9]. Numerous advantages have been attributed to computerized cognitive tests, including standardized and consistent administration procedures, precise control of stimulus presentation, capability to measure response times with high accuracy, automatic presentation of performance, both as raw scoring and normed results considering sociodemographic characteristics, ability to generate alternative forms appropriate for repeated testing, increased accessibility in remote locations, ability to develop large and accurate databases, and also reduced costs with personnel [8–12]. Nevertheless, some disadvantages can also be identified, such as cost of equipment and software, potential inaccuracies associated with uncontrolled testing environments, limitations for individuals with no computer familiarity or inability to use digital technology, lack of assessment flexibility, difficulty assessing speech disorders and behavioral deficits, and lack of proper validation [9].

Over 15 computerized cognitive tests for older adults have been identified [9], including full evaluation test batteries (e.g. the *Cambridge Neuropsychological Test Automated Battery* (CANTAB) [13]), as well as screening tests (e.g., *CNS Vital Signs* [14]; and *CogState* [15]), and short screening tests (e.g. CANTAB mobile [Cormack et al., 2018, Alzheimer’s &amp Dementia, 14(7S_Part_9), P522–P523]). Technological features vary between tests according to the hardware required (computer or tablet), mode of input (mouse, keyboard or touch screen) and mode of administration (examiner- or self-administered) [9]. Most studies include sufficient data concerning discriminant validity for Mild Cognitive Impairment (MCI) and dementia, with good to excellent accuracy capacities. However, results regarding other psychometric properties are not as well documented and data on concurrent validity with standard pencil-and-paper tests are inconclusive [9, 16, 17]. A more recent systematic review indicated that most digital instruments have comparable criterion validities with the paper-and-pencil tests and revealed good sensitivity and specificity for the detection of MCI or dementia [18]. Nevertheless, some limitations for the available studies were also underlined, including small sample sizes, lack of psychometric validation, and the existence of several tests without supporting data in a peer-reviewed publication [18].

The Cognitive Functions Dementia (CFD) [19] is a neuropsychological battery, part of the Vienna Test System (SCHUHFRIED GmbH, 2020) and aims to digitally assess the cognitive ability of people over the age of 50, especially in the context of neurodegenerative diseases. It is available in two parallel test forms so it can be alternatively employed in follow-up assessments. The CFD was developed to include cognitive domains and subdimensions covered by the DSM-5 diagnostic criteria for NCD, namely complex attention, executive functions, learning and memory, language and perceptual motor function. Social cognition is not assessed. Studies of the CFD include the original German version, as well as adaptations to Italian and English, but have not been published. The present study, using the Portuguese version of the test, aimed at examining psychometric properties of the CFD battery, namely construct and convergent validities, and reliability, as well as confirming its appropriateness to identify cognitive impairment, by analyzing criterion validity for NCD.

## MATERIALS AND METHODS

### The Cognitive Functions Dementia (CFD) battery

The CFD is a digital cognitive battery assessing five cognitive domains of the DSM-5 diagnostic guidelines for NCD. It was originally developed in German for people over 50 years of age, with samples from Germany, Austria and Switzerland, and has an estimated time of administration of 60 minutes. The proposed structure of the CFD battery includes assessment of 5 dimensions (complex attention, executive functions, learning and memory, language, and perceptual motor function) jointly describing an overall factor, the total CFD-Index. Primary variables from seven individual subtests included in the battery compose each of the dimensional indexes, as described below.

The Vienna Verbal Fluency Test [WIWO; Short names are always based on their German test name (e.g., WIWO = WIener WOrtflüssigkeitstest)] assesses both semantic and lexical verbal fluency, with respondents asked to name as many words as possible that belong to a certain category (semantic verbal fluency) or that start with a certain letter (lexical verbal fluency), within two minutes. The main variable is verbal fluency, which corresponds to the total number of correctly named words. Further information such as the number of rule violations (i.e., incorrect first letter or category), repetitions of already named words, and the total number of errors (rule violation plus repetitions) is stored.

The Vienna Object Naming Test (WOBT) assesses object naming with respondents asked to name different objects shown on the screen, one at a time. Lexical (the first letter of the target name) and/or semantic cues (description of the target object without naming the target word) are given if the subject is not able to spontaneously name the object. The main variable scores the total number of correctly named objects without cueing.

The Auditory Word List Learning Test (AWLT [20]) is a measure of learning ability, short-term and long-term verbal memory. Subjects are asked to learn a list of 12 words over four learning trials, with as many words as possible freely reproduced immediately after each learning trial, in a short-delay recall trial (5 minute break) and a long-delay recall trial (20-minute break). It also includes a recognition task, done immediately after the long-delayed recall trial, where subjects are asked to identify the 12 words from the list and reject 12 new words. Scoring main variables consist of *learning total*, *short-term delayed recall*, *long-term delayed recall* and *recognition.*

In the Perception and Attention Functions – Subtest Alertness (WAF-A) test, attentional alertness is assessed in three subtests. WAF intrinsic alertness is administered at the beginning of the assessment and measures the reaction time in response to simple visual stimulus material (a black circle on a white background). WAF intrinsic alertness retest is a presentation of the WAF intrinsic alertness at the end of the assessment, to measure fatigue by comparing the mean reaction times between both subtests. WAF phasic alertness with acoustic cueing is a version given with acoustic cues to examine whether reaction time benefits from presentation of the cue. The Perception and Attention Functions – Subtest Divided Attention (WAF-G) is a cross-modal (visual/auditory) measure of divided attention, where the respondent receives visual and auditory stimuli and is asked to determine whether one of the target stimuli (square or high tone) changes twice in succession. In all WAF tests and subtests, the main reported variables are *mean reaction times*, which are computed as logarithmic means (excluding responses < 100ms).

The Trail Making Test – *Langensteinbach* Version (TMT-L) is divided in two parts. Part A assesses processing speed by asking participants to link circles containing the numbers 1 to 25 in ascending order as quickly as possible. Part B measures cognitive flexibility by requiring subjects to alternately link circles containing the numbers 1 to 13 and the letters A to L in ascending order. The time needed to complete is the main variable for each part. TMT-L is not simply a digital version of the traditional TMT. It has several advantages including adjusting the layout of parts A and B so that the paths and the number of distractors are the same, running very similar numbers of items in the left and right directions to avoid long saccades, and enabling a completely standardized and automatized time measurement by establishing accurate starting and ending measurements.

The CORSI Block-Tapping Test (CORSI) is a measure of spatial working memory, where respondents are required to tap the blocks shown on the screen in reverse order than the one done by a hand icon that moves about the screen. The length of the sequences increases over the course of the test and terminates as soon as three successive sequences have been incorrectly tapped. The main variable for scoring considers the total number of blocks correctly tapped.

The Visuoconstruction Test (VISCO) is a measure of visuocontruction of perceptual-motor functioning where respondents are required to assemble a target shape made up of several equilateral triangles, by using triangles pointing upwards and downwards, within 60 seconds. The main variable reported is the total of correct responses, where two points are given for correct reproductions in the first 30 seconds, and one point if correctly finished in the last 30 seconds.

The battery was specially developed for older individuals as it offers specific features including test supervisor supported instructions (to account for the needs of people with very little or no experience with computers), a touchscreen operation (to avoid mouse and/or keyboard use), and audio recording for posterior editing in speech-based tests. It is also available in two parallel forms so that it can be alternatively employed in follow-up assessments. The two CFD forms comprise parallel versions of the subtests WIWO, AWLT, TMT-L, WOBT and VISCO to avoid practice effects between follow-up assessments.

Good psychometric properties for the CFD battery on a Germanophone norming sample of healthy volunteers are reported in the CFD test manual [19], available when the test is acquired. In that sample, a structural equation model indicated a satisfactory model fit [χ^2^(73) = 186.5; *p* <.001; CFI = 0.946; RMSEA = 0.062 (95 % Cl: 0.051; 0.073); SRMR = 0.07], with most items presenting adequate goodness-of-fit indices of local adjustment in a five-factorial second-order model. The five *a priori* defined cognitive functional areas were confirmed (attention, verbal long-term memory, expressive language, executive functions, and perceptual motor functions) as well as the combined CFD-Index. The weighted omega coefficient supported good to excellent reliability values in all dimensions, namely expressive language (0.7), attention (0.74), executive function (0.85), perceptual motor functions (0.93), verbal long-term memory (0.95), and the combined CFD-Index (0.91). The reliability of the individual tests was also computed for both test forms, with Cronbach’s alpha for most subtests reaching values above 0.7. The study manual also reports data for criterion validity of the CFD, in identification of major and minor NCD in convenience samples of patients previously diagnosed with Alzheimer's dementia (AD) or mild cognitive impairment (MCI), respectively. The combined CFD-Index exhibited, as expected, higher accuracy values for AD (sensitivity = 93.8%, specificity = 87.5%) than for MCI (sensitivity = 73.5%, specificity = 88.2%) when compared to healthy participants from the norming sample. In this clinical sample, individual tests of the CFD battery had Cronbach’s alpha values above 0.7 for all tests except the AWLT recognition (0.69).

### Procedures

#### Translation and adaptation

The CFD employed in this study was version 8.10 (Vienna Test System, version 8.10.11.33957). CFD test materials, namely those including verbal components, such as the WIWO and AWLT, were adapted to European Portuguese by a linguist specialized in the adaptation of cognitive instruments (CM). Translation of test instructions was performed by a translation services agency.

In the WIWO lexical subtest two letters were chosen based on the most and second most common initial letters of words present in two European Portuguese databases, namely *Procura-PALavras* (P-PAL; http://p-pal.di.uminho.pt/about/project?lang=en) and *Léxico Multifuncional Computorizado do Português Contemporâneo* (CORLEX; www.clul.ulisboa.pt/en/10-research/735-multifunctional-computational-lexicon-of-contemporary-portuguese). Both resulted in identification of *C* as the most common initial letter, used in the test form 1, and *A* as the second most common initial letter, used in the test form 2. For the semantic word fluency task, since no reasons were identified to proceed otherwise, the original categories were maintained, namely *animals* for test form 1 and *first names* for test form 2.

The AWLT adaptation considered words according to the linguistic and cultural adequacy criteria recommended by the original authors [20], including orthographic neighborhood size (normalized neighborhood size between 5 and 15), frequency (normalized frequency between 5 and 15) and length (only mono- or disyllabic words), and the semantic content (12 categories: furniture, food, transportation, clothing, tools, recreation, animals, plants, buildings, musical instruments, kitchen, and daily life). All words needed to be nouns and easily imaginable. The P-PAL linguistic database provided information regarding all the required formal criteria, and a first list of lemmas (word types) was generated meeting all the formal criteria. Since four lemmas were not available for all semantic categories, although meeting the remaining criteria, the range of neighborhood size and frequency was widened, with items and distractors carefully distributed between forms 1 and 2, so as to obtain similar values, both in maximal range and in mean frequency and neighborhood size for each of the lists. The adaptation process resulted in two final lists of 12 words and 12 distractors.

#### Administration procedures

All participants underwent a research protocol including a sociodemographic and clinical questionnaire, the CFD battery, the Geriatric Depression Scale – 15 items (GDS-15) [21], the Montreal Cognitive Assessment (MoCA) [5, 22] and a brief satisfaction questionnaire. CFD testing was conducted exclusively in a one-on-one setting, with the participant and a test supervisor. Convertible laptops (Lenovo Yoga 700-14ISK and Lenovo Yoga 520-14IKB) were used as recommended by the test manufacturer. The laptops had 14-inch touchscreens that could rotate 360 degrees and could therefore be operated like tablets. Test supervisors did not have formal neuropsychology training but received brief training to become familiar with the CFD and with the test manual. Participants in the healthy sample attended two testing dates. While these were planned to be approximately 30 days apart, Covid-19 pandemic contingencies imposed delays between the two sessions for some participants. CFD form S1 and all remaining questionnaires were administered during the first visit. During the second session only the CFD form S2 was presented. Study procedures and protocol were reviewed and approved by the Ethics Committees of the Champalimaud Foundation and Centro Hospitalar de Lisboa Ocidental. Informed consent was obtained from all participants, and the study was conducted in accordance with the Declaration of Helsinki. Data was collected, stored, and processed in accordance with ethical principles and applicable international, EU and National legislation.

### Participants

Healthy participants for the norming sample were native Portuguese speakers, 50 years of age or older, recruited in the community using notices placed on noticeboards in organizations for older adult and senior education centers, or online via social media, between 2018 and 2022. A predefined stratified quota plan was used considering a balanced distribution according to sex, age (50+), and educational level, across a range of geographic locations including the metropolitan areas of Lisbon, Setúbal, Porto, Portalegre, and Coimbra. The quota plan aimed to achieve an even distribution of participants with specific combinations of sex, age and education. Participants were excluded according to the following criteria: illiteracy; diagnosis of any neurological or mental illness within the past year; radiotherapy or chemotherapy within the past year; severe head injury, concussion, encephalitis, or stroke in the course of life; confusion or loss of consciousness within the last five years, including in the course of hospitalization. Participant data was also excluded when GDS-15 scores were greater than 10, suggesting moderate to severe depression [21, 23]. Additional criteria were used for exclusion of individual test session data, namely: manual cancellation of running test; duplicate cases; implausible values with test results and response patterns indicating testing behavior non-compliant with the test instructions in 2% or more data per test session; missing data. Patients were recruited for criterion validity estimation, according to previous diagnosis of mild NCD (MCI) or major NCD (dementia) following international consensus diagnostic criteria (DSM-5 criteria) [2]. Patients were recruited during routine neurology or psychiatry visits to the Champalimaud Clinical Center or Centro Hospitalar de Lisboa Ocidental.

### Data analysis

Sociodemographic and performance data are presented as means and standard deviations (SD). Primary analyses concerned structural validity of S1 data according to the predefined theoretical model, assessed by means of confirmatory factor analyses (CFA). Measurement invariance of the assumed model with respect to the original Germanophone norming sample was assessed by means of multi-group confirmatory factor analysis (mgCFA). Model fit indices included: (a) nonsignificant χ^2^; (b) the comparative fit index (CFI) and the Tucker–Lewis index (TLI), with absolute indexes ≥ 0.90 indicating good fit and values ≥ 0.95 indicating very good model fit; and (c) the Root Mean Square Error of Approximation (RMSEA), and the Standardized Root Mean Square Residual (SRMR) indexes with values ≤ 0.08 indicating an acceptable fit and values ≤ 0.05 indicating a very good model fit [24, 25]. For model estimation, the variances of all latent variables were fixed to 1, while the factor loadings and variances of all main variables (including VISCO) and factor loadings of the latent variables were freely estimated. Robust goodness-of-fit indices were used to evaluate the fit of the model [26-28]. Moreover, we provide results for structural relations in terms of intercorrelations across all dimensions and tests of the battery for both sessions. Internal consistency was estimated by means of Cronbach’s alpha for all test variables in both test sessions, and the weighted McDonald’s omega coefficient for the dimensional indices and cross-dimensional index [29]. Re-test reliability was assessed using the intraclass correlation coefficient (ICC) with a two-way mixed effects model where individual effects are random and measures effects are fixed (type: absolute agreement). ICC values between 0.5 and 0.75 indicate moderate reliability, between 0.75 and 0.9 indicate good reliability, and > 0.90 indicate excellent reliability [30]. To assess convergent validity, we used Pearson correlation coefficients between the CFD-Index and the MoCA scores.

To assess criterion validity, receiver operating characteristics (ROC) were calculated for the CFD-Index and dimension-specific indices. The accuracy of each index in predicting group membership (cognitively healthy versus NCD) was estimated through the area under the curve (AUC), with larger values indicating better accuracy. The optimal cutoff points were calculated for each group according to the highest Youden index, which indicates the cutting score that maximizes the sensitivity and specificity of the classification process. This analysis was implemented in MedCalc (version 20.218; MedCalc Software, Ostend, Belgium).

Other statistical analyses were performed using the Statistical Package for the Social Sciences (SPSS, Version 28.0; IBM SPSS, Inc., Chicago, IL), with the “lavaan” R package, Version 0.6-12 (R Core Team, 2016; Rosseel, 2012) used for the Structural Equation Model definition. Results with an alpha-level (*p*) < 0.05, two-sided, were considered statistically significant, and the Bonferroni alpha correction was used when applicable. In group comparisons, whenever assumption of homogeneity was violated, a Welch-test was performed.

**Figure 1.**
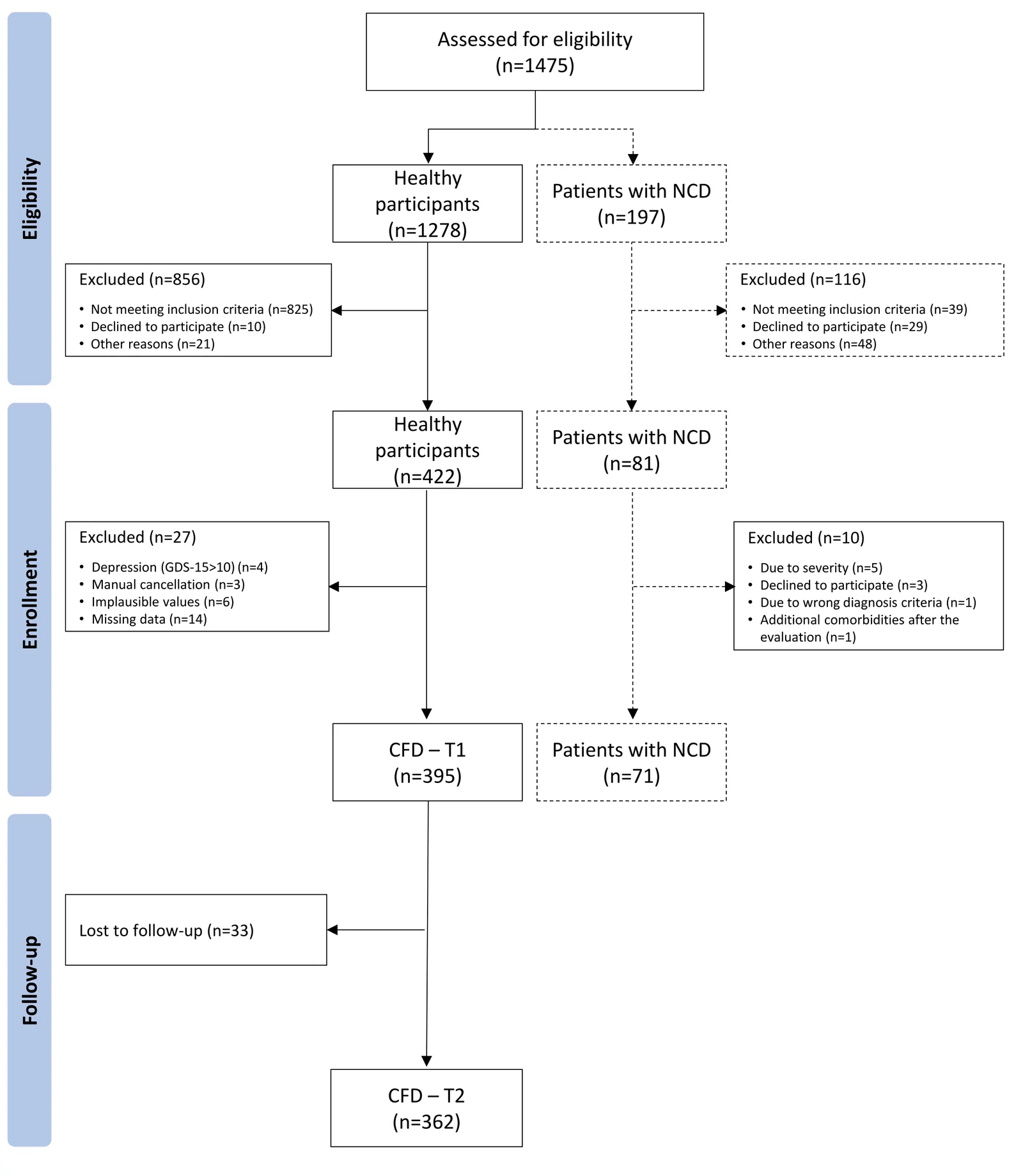
**Flow diagram of the study enrollment and follow-up process.** NCD – Neurocognitive Disorder; GDS-15 – Geriatric Depression Scale (15 items); CFD – Cognitive Functions Dementia; T1 – First session; T2 – Re-testing session. Exclusion criteria descriptions are as following: Depression score – GDS-15>10; manual cancellation - existence of manually cancelled test runs; implausible values – test results and response patterns that indicate testing behaviour non-compliant with the test instructions (e.g.: extremely short working time; constant same response); missing data – missing values in the main variables.

## RESULTS

### Norming sample

For the norming sample of cognitively healthy participants, we assessed 1,278 people, 422 of whom were eligible and within the required norming groups and thus recruited. After testing this sample, some participants were excluded due to ineligibility, invalid data or incomplete data, resulting in a total of 395 complete cases across all tests in the first session, which was the final norming sample (CFD – S1) (Table 1). This sample had 179 males and 216 females, with a mean age (±SD) of 65.93 (±9.80) years; and higher frequency of participants with secondary (n=116) and university (n=108) educational degrees. For the re-testing session, there were a total of 372 complete cases across all tests (CFD – S2), with the merge across both sessions resulting in 362 complete cases for re-test analyses. The time interval between S1 and S2 sessions ranged between 15 and 115 days (mean=30.54, median=28, SD=12.99). Demographic information of the norm samples S1 and S2 is presented in Table 1.

**Table 1.** Demographic and clinical characteristics of the final CFD norming samples in the first session (T1) and in the re-testing session 2 (T2).

	CFD – T1 (N=395)	CFD – T2 (N=362)

**Age, mean (SD)**	65.93 (9.80)	65.82 (9.81)
**[Min – Max]**	[50 - 91]	[50 – 91]

**Male sex, n (%)**	179 (45.3)	166 (45.8)

**Educational level, n (%)**		
**1 (≤ 4 years)**	81 (20.5)	73 (20.2)
**2 (5 – 6 years)**	40 (10.1)	40 (11.0)
**3 (7 – 9 years)**	116 (29.4)	108 (29.8)
**4 (10 – 12 years)**	50 (12.7)	45 (12.4)
**5 (≥ 13 years)**	108 (27.3)	96 (26.5)

**MoCA, mean (SD)**	22.66 (4.05)	--
**[Min – Max]**	[9 – 30]	

**GDS, mean (SD)**	2.49 (2.37)	--
**[Min – Max]**	[0 – 10]	

*Note*: MoCA – Montreal Cognitive Assessment; GDS – Geriatric Depression Scale

### Construct validity

CFD operationalization, with selection of appropriate test paradigms to measure specific cognitive subdimensions, was previously defined, based on the potential value of these paradigms to assess domains covered by the DSM-5. Descriptive statistics of individual CFD test scores across both test forms are depicted in Supplementary Table 1.

#### Structural validity

**Figure 2.**
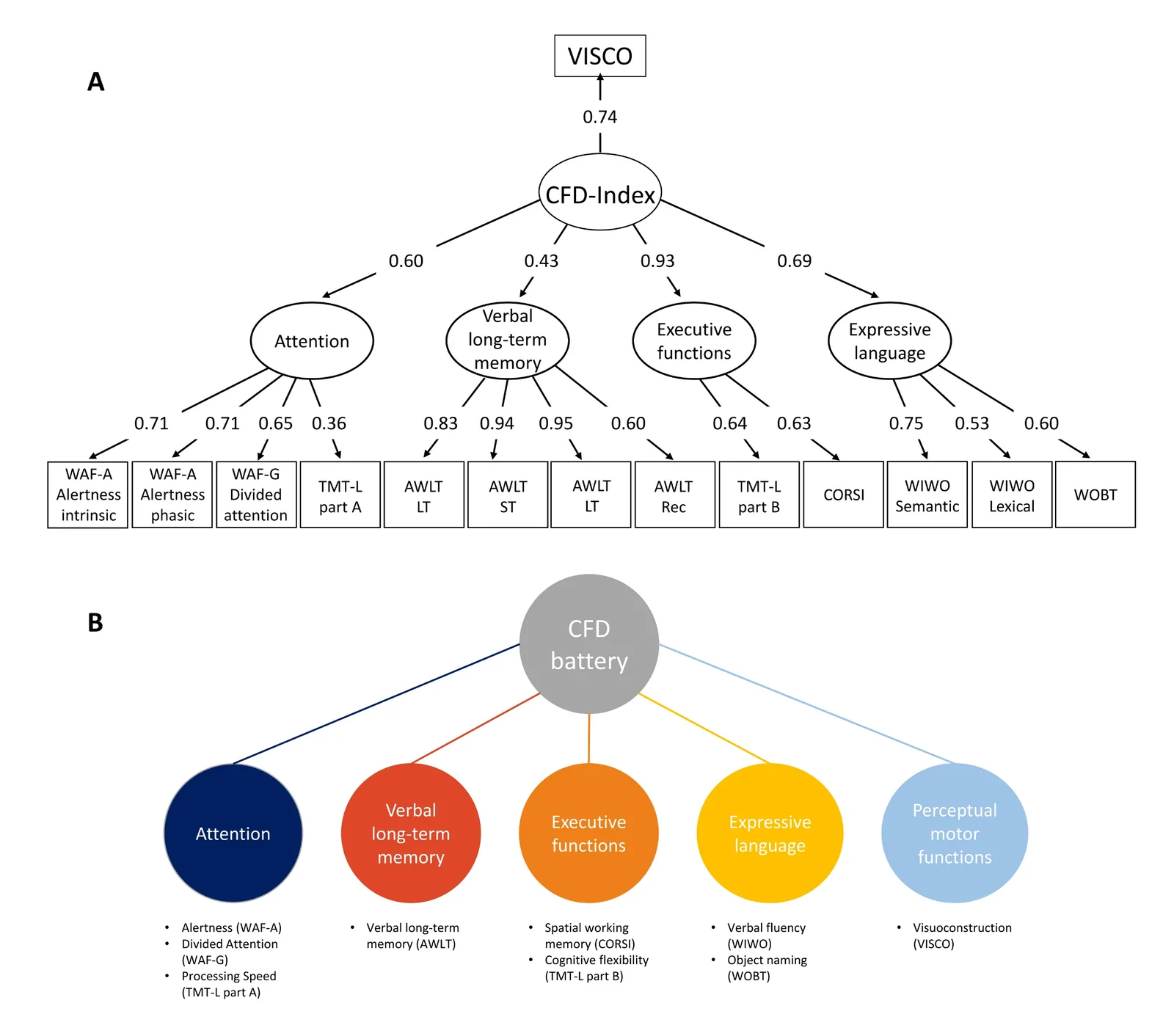
**Cognitive Functions Dementia (CFD) battery. (A)** Model with factor loadings used to test measurement invariance. **(B)** Theoretical model with five dimensions and nine individual tests. WAF: Perception and Attention Functions; TMT-L: Trail-Making Test – *Langensteinbach* Version; AWLT: Auditory Word List Learning Test; LT: Learning total; ST: Short-term delayed recall; LT: Long-term delayed recall; Rec: Recognition; CORSI: CORSI Block-Tapping Test; WIWO: Vienna Verbal Fluency Test; WOBT: Vienna Object Naming Test; VISCO: Visuoconstruction Test.

A confirmatory factor analysis (CFA) on data from the first test session was conducted to assess whether the proposed model [19], with the several subtests, five cognitive domains (attention, verbal long-term memory, expressive language, executive functions, and perceptual motor functions) and the general factor CFD-Index (Figure 2a), could be generalized to the present CFD norming sample. Prior to the primary analysis, the main variables of the tests included in the CFD were z standardized. For the main variables which indicate reaction times (WAF and TMT-L), polarity was reversed, so that for all variables higher values indicate higher ability. Some further cases with extreme scores (multivariate outliers) in the TMT-L and WIWO lexical main variables, leading to non-convergence of the model, were excluded before conducting the CFA, resulting in a sample size of 372. The Mardia’s test of multivariate skew and kurtosis revealed lacking multivariate normality, with the null hypothesis being rejected (p < 0.001), which was confirmed by the Q-Q-plot [31], leading to the maximum likelihood estimation with robust standard errors and multilevel modelling (MLM) Satorra-Bentler scaled test statistic being used in the CFA [32, 33]. Overall, the model showed a satisfactory fit, χ^2^(73) = 227.21; *p* < 0.001, CFI = 0.94, TLI = 0.92, RMSEA = 0.08 (95 % Cl: 0.07; 0.09), SRMR = 0.06 [24, 25], confirming the validity of the original specified structure of the model. Furthermore, correlations between tests (Supplementary Table 3 - first testing session; Supplementary Table 4 - re-testing session) were in line with the theoretical model.

#### Measurement invariance

We assessed measurement invariance across the Germanophone and the present norming sample by means of multi-group confirmatory factor analyses (mgCFA) [25]. With mgCFA, an initial baseline model is established, assuming configural invariance, where only equal factor structure is required – with the same number of factors and same indicators per factor - and all other parameters are estimated freely. Subsequently, these parameters are restricted stepwise towards strict invariance [34]. Here, the baseline was specified by the measurement model derived from the Germanophone norming sample with its specified factor structure (configural invariance) and factor loadings (metric invariance, where factor loadings are required to be equal across groups; see Fig. 2A). Overall, this baseline model had an acceptable fit to the data from our sample (CFI = 0.91, TLI = 0.91, RMSEA = 0.08 [95 % Cl: 0.08; 0.09]), supporting metric invariance [24, 25]. For the more restrictive strong invariance, which constrains equality of intercepts across groups, the difference in the fit indices (ΔCFI = -0.03 and ΔTLI = -0.03) exceeded the recommended difference of -0.01 [35]. Therefore, the analysis was concluded, and strict invariance (further imposing cross-group equality restrictions on residual invariances) was not assessed [25]. Table 2 shows the fit indices for all nested models and further illustrates that a configural model with unconstrained factor loadings is equal to a metric model with equality-constrained factor loadings.

**Table 2.** Summary of the nested multi-group confirmatory factor analyses (mgCFA) models.

Test	N	χ2	*p*	df	CFI	TLI	RMSEA
**Configural invariance**	779	596.97	<0.001	172	0.91	0.91	0.08
**Metric Invariance**	779	596.97	<0.001	172	0.91	0.91	0.08
**Strong invariance**	779	768.58	<0.001	180	0.88	0.88	0.09

*Note:* df – degrees of freedom; CFI – comparative fit index; TLI – Tucker–Lewis index; RMSEA – Root Mean Square Error of Approximation.

### CFD-battery indices

The five dimension-specific indices, and the cross-dimensional CFD-Index, represent the main variables of the CFD battery, as depicted in Figure 2b. They were calculated based on the factorial structure and weighted by the factor loadings of the CFA-analysis. For calculation of dimension-specific indices, attention considered WAF-A, WAF-G and TMT-L; verbal long-term memory corresponded to AWLT main variables; executive functions included TMT-L B and CORSI; expressive language the WIWO and WOBT; and perceptual motor function corresponded to VISCO. The cross-dimensional CFD-Index was aggregated across all dimension-specific indices and weighted according to the higher factor loadings. The values of test main variables were z-standardized based on the mean and standard deviation of the original norm sample, not controlling for sociodemographic variables. Additionally, for the main variables which indicate reaction times (WAF and TMT-L) polarity was reversed, so that for all variables higher values indicate higher ability. Descriptive statistics can be seen in Supplementary Table 2.

### Reliability

Reliability results for specific tests were good to excellent for most test variables (Supplementary Table 5). Only for the WIWO semantic and the AWLT delayed recall and recognition tests did we observe Cronbach’s alpha coefficients below 0.7. Similarly, for re-test reliability (ICC), verbal fluency – semantic (WIWO: ICC = 0.68), AWLT recognition (ICC = 0.70), and WAF-A mean reaction time – intrinsic (ICC = 0.73) were the only tests that did not reach beyond moderate reliability. We also analyzed the mean differences between first (T1) and second (T2) assessments and found a significant improvement for WIWO semantic [*t*(361) = -7.59, *p*<0.001] and VISCO [*t*(361) = -4.35, *p*<0.001], and a significant decrease for WIWO lexical [*t*(361) = 3.82, *p*<0.001]. These effects were small [36], except for the WIWO semantic, with a medium effect size (Supplementary Table 6). Since the use of parallel test forms allowed for control of learning effects, the most plausible explanation is familiarity with the tasks and the instructions.

For the indices, on the other hand, reliability assessment was good to excellent. Weighted omega coefficients were in the good to excellent range, between 0.69 for expressive language to 0.95 verbal for long-term memory. The coefficients for re-test reliability varied between 0.87 for executive functions and 0.95 for the cross-dimensional CFD-Index, favoring excellent temporal stability, namely for the main index. Sensitivity analyses confirmed that the uncontrolled interval between assessments (within vs. beyond 30 days) had no impact on participants' performance across the different indices (Table 3).

**Table 3.** Coefficients for weighted Omega (Ω) and re-test (ICC) reliability for all dimension-specific indices and the CFD-Index.

Index	*Omega (Ω)*	*Re-Test (ICC)*
Attention	0.74	0.89
Verbal long-term memory	0.95	0.89
Expressive language	0.69	0.91
Executive functions	0.85	0.87
Perceptual motor function	0.94	0.93
**CFD-Index**	**0.91**	**0.95**

Note: ICC – intraclass correlation coefficient

Sensitivity analyses were performed to confirm whether the uncontrolled interval between assessments could have an impact on participants' performance across the different indices. Similar ICCs were found between the group retested within 30 days (N = 279) and those retested beyond 30 days (N = 83) on any index (Attention index: 0.89 and 0.89; Verbal long-term memory index: 0.87 and 0.92; Expressive language index: 0.92 and 0.87; Executive functions index: 0.87 and 0.90; Perceptual motor index: 0.93 and 0.91; CFD-Index: 0.95 and 0.96, respectively). Moreover, no differences were found in performance at retest between the two groups on any index (independent sample t-tests; Attention index: *p*=0.87; Verbal long-term memory index: *p*=0.4; Expressive language index: *p*=0.52; Executive functions index: *p*=0.6; Perceptual motor index: *p*=0.3; CFD-Index: *p*=0.64).

### Convergent validity

Pearson correlation analysis was performed between the CFD-Index and the MoCA test total score to obtain convergent validity measurements. The CFD-Index was selected for this analysis as it provides a general cognitive function measurement, similar to MoCA. Results showed a significant positive correlation (r = 0.77, *p *< 0.001) between the two measures, supporting that they both measure general cognitive functioning.

**Table 4.** Differences for CFD indices between sex, education and age levels.

Test	Variable	*Sex (males vs. females)*					*Education (levels 1-3 vs. 4-5)*					*Age Younger [50-64 years] vs. older elders [≥65 years]*				
		*t*	*df*	*sig.^b^*	*mean difference*	*Cohens’ d*	*t*	*df*	*sig. ^b^*	*mean difference*	*Cohens’ d*	*t*	*df*	*sig. ^b^*	*mean difference*	*Cohen’s d*
**CFD-indices**	CFD-Index	1.80	393	0.07	0.82	0.18	-10.38	393	<0.001*	-4.27	-1.07	6.14	393	<0.001*	2.67	0.62
	Attention	3.11	393	0.002*	0.48	0.32	-8.57^a^	376.65	<0.001*	-1.21	-0.85	3.26	393	0.001*	0.50	0.33
	Verbal long-term memory	-1.71	393	0.09	-0.51	-0.17	-4.32	393	<0.001*	-1.28	-0.44	7.58^a^	376.56	<0.001*	2.10	0.76
	Expressive language	1.39	393	0.17	0.24	0.14	-11.26	393	<0.001*	-1.73	-1.16	2.78	393	0.006	0.48	0.28
	Executive functions	1.62	393	0.11	0.27	0.16	-7.97^a^	392.80	<0.001*	-1.20	-0.76	5.62^a^	355.76	<0.001*	0.92	0.57
	Perceptual motor function	4.32^a^	359.6	<0.001*	0.44	0.44	-9.17^a^	284.03	<0.001*	-0.91	-0.98	3.59^a^	384.74	<0.001*	0.36	0.36

*Notes:* Education levels: 1 (≤ 4 years); 2 (5 – 6 years); 3 (7 – 9 years); 4 (10 – 12 years); and 5 (≥ 13 years).

Positive mean difference indicates higher scores for male participants, for participants with education level 1-3, and for younger participants (50-46 years).

a Violation of homogeneity assumption -> Welch-test.

b The alpha level was Bonferroni corrected (α = 0.0025), significant p-values are marked with an*.

### Group differences

Group differences in indices for the first test session were assessed for sex, age, and education level, using t-tests for independent samples, or Welch-test if the assumption of homogeneity was violated. For all indices, except for perceptual motor function, there were no differences according to sex. However, participants with education levels 1-3 (≤9 years: 237 individuals) performed worse than those with education levels 4-5 (≥10 years: 158 individuals) across all indices, with medium to large effect sizes (Cohen’s *d* -1.16 to -0.44), while those 65 years or younger (198 participants) performed better than those older than 65 (197 subjects; median split), with small to medium effects (Cohen’s *d* 0.28 to 0.76; Table 4). To further assess the effects of age, Pearson correlations were conducted with CFD-indices, revealing significant negative correlations for all indices (-0.39 < *r* < -0.21, *p* < 0.001), further supporting better performance for younger participants. Group differences in the test main variables were also assessed (Supplementary Table 7).

### Criterion validity

The CFD battery was developed to detect cognitive impairment according to the DSM-5 diagnostic criteria for NCD. We thus aimed at analyzing criterion validity for the CFD, i.e., examining sensitivity and specificity in identifying NCD [2]. To that effect, we recruited 71 patients, including 45 patients with mild NCD (MCI), and 26 with mild to moderate major NCD (18 with Alzheimer’s dementia, 4 with frontotemporal dementia, and 4 with Lewy body dementia). In this population, the CFD-Index and MoCA test scores were positively correlated (r = 0.77, *p *< 0.001) similarly to what was found in the full norming sample and further supporting convergent validity of the CFD. To assess criterion validity, a group of healthy participants from the norming sample (n= 196) was frequency matched to age, sex, and education level of the patient sample [37].

**Table 5.** Sociodemographic and clinical characteristics of the clinical group (NCD) and the healthy participants.

	Clinical group (NCD) (n=71)	Healthy group (n=196)

**Age, mean (SD)**	72.83 (7.41)	70.94 (7.82)*^NS^*
**[Min – Max]**	[55 – 88]	[51 - 88]

**Sex, male | female**	25 | 46	75 | 121*^NS^*

**Educational level, n**		
**1 (≤ 4 years)**	16	35
**2 (5 – 6 years)**	1	2
**3 (7 – 9 years)**	14	57
**4 (10 – 12 years)**	10	19
**5 (≥ 13 years)**	30	83 *^NS^*

**MoCA test, mean (SD)**	17.71 (5.15)	22.81 (4.41)^*^

**GDS, mean (SD)**	5.07 (3.90)	2.55 (2.39)^*^

*Notes:* Comparisons between the sociodemographic variables of the two groups were carried out by an independent sample t-test for age, and chi-squared test for sex and education. MoCA – Montreal Cognitive Assessment, GDS – Geriatric Depression Scale. *NS* – non-significant;

* *p* < 0.001

As expected, while sociodemographic characteristics of the NCD and cognitively healthy groups were not significantly different regarding age [*t*(265) = -1.77, *p* = 0.08], sex [*χ*^2^ (1) = 0.21, *p* = 0.65] or education level [*χ*^2^(4) = 3.31, *p* = 0.51], the NCD group had significantly lower cognitive performance in the MoCA performance [*t*(260) = 7.78, *p* < 0.001] and more severe symptoms of depression as assessed by the GDS [*t*(263) = -6.32, *p* < 0.001] (Table 5).

CFD indices were significantly lower in the NCD group, with medium to large effect sizes, particularly the cross-dimensional CFD-Index and the dimension-specific indices for attention, verbal long-term memory, expressive language, and executive functions. ROC curve analyses, assessing criterion validity of CFD indices in identifying NCD, showed that all indices had significant AUCs, with verbal long-term memory (AUC=0.81), CFD-Index (0.79), attention (0.77) and expressive language (0.76) being the most accurate, and executive functions (0.67) and perceptual motor functions (0.62) being the least accurate in distinguishing the two groups (Table 6). Exploratory analyses separating analyses for mild NCD/MCI and major NCD/dementia can be found in Supplementary Tables 8 and 9.

**Table 6.** CFD indices differences between the clinical group (NCD) and the healthy participants and diagnostic classification accuracy in discriminating the groups.

	Clinical group (NCD) (n=71)	Healthy group (n=196)	*t test*	df	*sig.^b^*	Cohen’s d	AUC	95% CI	*sig.*	Sensitivity	Specificity
CFD-Index	-9.60 (6.33)	-3.31 (4.82)	7.60^a^	100.9	<0.001*	1.19	0.79	[0.74, 0.84]	<0.0001	87.32	57.65
Attention	-2.56 (2.70)	-0.28 (1.49)	6.70^a^	84.5	<0.001*	1.21	0.77	[0.71, 0.82]	<0.0001	62.86	82.14
Verbal long-term memory	-5.61 (3.71)	-1.15 (3.09)	9.05^a^	107.3	<0.001*	1.36	0.81	[0.76, 0.86]	<0.0001	66.20	85.71
Expressive language	-3.63 (1.90)	-1.86 (1.75)	7.07	264	<0.001*	0.98	0.76	[0.71, 0.81]	<0.0001	72.86	71.43
Executive functions	-2.12 (2.56)	-0.94 (1.92)	3.31^a^	82.1	<0.001*	0.56	0.67	[0.60, 0.72]	<0.0001	70.49	59.69
Perceptual motor functions	-0.96 (0.97)	-0.64 (0.98)	2.30	258	0.02	0.33	0.62	[0.56, 0.68]	0.005	54.69	68.88

*Notes:* Data are expressed as mean (SD). Comparisons between the two groups were carried out by independent sample t-tests.

a Violation of homogeneity assumption -> Welch-test.

b The alpha level was Bonferroni corrected (α = 0.008), significant p-values are marked with an*. AUC – area under the curve; CI – confidence interval. Sensitivity and Specificity values are expressed in percentage.

## DISCUSSION

The Cognitive Functions Dementia (CFD) is a computerized battery developed to assess the cognitive domains of neurocognitive disorder (NCD) included in DSM-5 criteria, namely complex attention, executive functions, learning and memory, language, and perceptual motor function [2]. While the battery is commercially available based on data collected in a Germanophone sample, made available when the battery is acquired [19], data on norming and validation is yet to be peer-reviewed and published for the scientific community. Here we analyzed the psychometric properties and clinical validity of the CFD for NCD. Overall, the CFD was found to have good psychometric properties in a population of cognitively healthy individuals older than 50 years, and also good criterion validity for diagnosis of NCD.

A five-factor second-order model structure confirmed the pre-defined functional cognitive areas, namely attention, verbal long-term memory, expressive language, executive functions, and perceptual motor functions, as well as an overall factor, the combined CFD-Index. These results confirm that the structure of the model specified originally was valid for our norm sample. Assessment of measurement invariance was considered to analyze if model specifications derived from the Germanophone sample of the CFD are valid for the present sample. Results showed that while invariance was tenable at the metric level, the difference in fit indices did not support strong factorial invariance [35], and thus strict invariance was not assessed [25]. Rejection of strong invariance suggests a differential additive response style, which is likely due to effects of culture, cohort, or data collection, resulting in a constant difference between groups that is unrelated to the factors that decrease or increase the overall level of performance [25]. As a result, this limits the direct comparability of scores across populations and further underscores the need for caution when applying the CFD in other (international) contexts. Nevertheless, our findings support that the model on which the calculation of the CFD indices is based is valid over both norming samples, confirming that the number of factors, their respective indicators, and the specific constrained factor loadings can be regarded as equal for the two samples, and establishing common constructs similarly manifested in both populations. Hence, the same weighting scheme, and specifically the index calculation of the original sample, can be applied to both norms. However, since strong invariance was not supported, it must be inferred that intercepts of the two samples differ, strongly supporting the use of separate norms for each population, in accordance with available guidelines [38].

Dimensionality findings were endorsed by reliability. For the specific test variables, Cronbach’s alpha was good to excellent for most cases, with the exception of WIWO semantic (semantic verbal fluency), and AWLT delayed recalls and recognition (verbal long-term memory) tests, with coefficients somewhat below 0.7, but within the limits of being considered adequate [39, 40]. While similar lower values were reported in the Germanophone sample [19], the asymmetry and kurtosis values for these variables are in agreement with a normal distribution [41], ruling out the possibilities of a floor or ceiling effect. Furthermore, from the CFA results, these tests had good loading values. Most importantly, when considering the six CFD indices, some of which include these tests, they had good to excellent reliability values, with Omega (Ω) ranging from 0.69 to 0.94. Test-retest reliability was good to excellent for most test variables, except for the WIWO semantic, the AWLT recognition, and the WAF-A mean reaction time, which had moderate values [30]. Test-retest reliability was excellent across all CFD indices. Unfortunately, in the present study, due to Covid-19 pandemic contingencies, the initially defined test-retest interval (30 days) could not be controlled for all participants. Yet, test-retest reliability was similar between the groups with different interval assessments (within and beyond 30-days), and no differences were found between the two groups on any CFD index. We acknowledge that this constraint requires cautious interpretation of test-retest reliability. Nevertheless, reliability was enhanced by the use of CFD indices, relative to the specific tests within CFD.

The CFD general index was compared to the widely used paper-and-pencil version of the MoCA test, to obtain a measure of convergent validity. The MoCA test is a validated measure of general cognitive functioning, extensively used as a screening instrument for cognitive dysfunction [5, 42]. A significant and high positive correlation between the CFD-index and MoCA score corroborated that both relate to general cognitive functioning and that the CFD compares well to a cognitive screening test requiring specific training and certification [43]. For the CFD, no certification is needed for the test mediator and, while the MoCA is a cognitive screening test, the CFD can be considered a neuropsychological battery, as per the American National Academy of Neuropsychology [6], enabling a more nuanced evaluation of specific cognitive domains, and thus supporting differential diagnosis. Convergent validity was similarly computed in the clinical sample, between the CFD general index and the MoCA test, with equivalent results. This further supports that the CFD compares well to a traditional paper-and-pencil assessment, even among patients with MCI or dementia, overcoming a limitation of many studies on computerized cognitive testing in older populations [17]. Indeed, in the CFD, test instructions are always accessible and can be shown or hidden at any time with the assistance of a mediator, who is available to clarify the instructions whenever needed. Also, for ethical reasons, this is crucial in the context of aging [17] and is in accordance with recommendations from the American Academy of Clinical Neuropsychology (AACN) and the National Academy of Neuropsychology (NAN) on Computerized Neuro-psychological Assessment [44]. The effectiveness of self-administered tests is limited in users who are not comfortable or proficient with technology, which is particularly relevant for older adults or individuals with limited experience using digital devices. Moreover, a misinterpretation of instructions may lead to errors that affect the results and undermine the validity of the test. In subsequent research, studies should assess the convergent validity of the CFD with traditional full neuro-psychological batteries, to enhance its clinical value and establish its utility beyond that of a screening tool.

Sociodemographic and individual factors such as sex, age, and education, among others, have important impact on the validity and reliability of psychological tests [45], including neuropsychological assessment. Here we addressed demographic moderators of cognitive function, across CFD indices and specific test variables. With regards to sex-dependent differences, male participants scored better in the attention and perceptual motor functions indices. The influence of sex in cognitive performance of older adults is not unanimous, with some studies supporting superiority for males [46-48], others for females [46, 47] and others supporting no differences [47, 49]. Differences may result from hormonal and genetic variations [50], brain structure and function [51-53], and social factors, such as role models, life experiences, and social expectations [54, 55]. Age, on the other hand, is a well-known moderator of cognitive function [38, 56, 57]. As expected, younger participants in our norming sample displayed better performance in the dimensional indices and test main variables, with small to medium effects. Furthermore, age was significantly correlated with the CFD-indices and the main test variables. WAF-A subtests, however, did not correlate with age, and did not differ when comparing younger and older elders, which is consistent with previous reports of age-related preservation of attention [48, 58]. Finally, and also as expected [54, 59], more educated individuals outperformed low educational groups in all indices and test main variables, with medium to large effect sizes. Overall, these findings highlight the importance of having specific norms that consider demographic variables, namely sex, age and education.

The CFD was developed specifically to address the DSM-5 criteria for NCD. Analysis of criterion validity for identification of NCD (mild NCD/MCI, and major NCD/dementia) was thus a critical objective of this work. We compared a clinical sample diagnosed with NCD to a frequency-matched subsample of cognitively healthy participants from the norming sample, to ensure that cognitive differences would not be better explained by sex, age or education. Not surprisingly, the clinical group had a significantly lower CFD performance, across all CFD indices, albeit this comparison not surviving the alpha correction for the perceptual motor functions index, possibly due to predominance of patients with amnestic MCI and AD, as well as frontotemporal dementia, who have preserved visuoconstruction abilities. Indeed, the largest effect size was for the verbal long-term memory index, expected in amnestic NCD. These findings are consistent with CFD data from the Germanophone sample, where significant differences were found in all CFD indices, but with perceptual motor functions index showing the lowest effect size in mild NCD [19]. ROC curve analyses confirmed these findings, with the perceptual motor functions index showing the smallest AUC, and the verbal long-term memory index the largest AUC, in differentiating NCD from cognitively healthy individuals. Importantly, the CFD-index, as a combined score, had a good discriminatory ability to identify NCD. While the clinical sample size in our study limits subsample analyses, exploratory analyses support superior discrimination of the CFD general index for major NCD (dementia) than for minor NCD (MCI), which is consistent with data from the Germanophone sample, reporting higher accuracy for AD (sensitivity = 93.8%, specificity = 87.5%) than for MCI (sensitivity = 73.5%, specificity = 88.2%) when discriminating from healthy participants [19]. Nevertheless, we acknowledge that our exploratory analyses separating mild NCD/MCI and major NCD/dementia should be interpreted with caution due to the small subgroup sizes, which prevent reliable subtype-level conclusions and may lead to inflated estimates of diagnostic performance. Moreover, the limitation of the small and predominantly Alzheimer's-focused clinical sample may affect the generalizability of the findings to other etiologies. Future studies should aim to validate the CFD across a broader range of clinical subtypes to enhance its applicability and robustness across diverse neurocognitive disorders.

Although no direct comparisons can be made between the CFD and other computerized batteries, we acknowledge comparable characteristics with some of these batteries, such as their application to older adults, use of technician-administered computerized formats, and relatively long administration times (typically exceeding 20 minutes). These include the *Automated Neuro-psychological Assessment Metrics* (ANAM) [60], the *Cambridge Neuropsychological Test Automated Battery* (CANTAB) [13], the *Cognitive Drug Research Computerized Assessment System for Dementia* (COGDRAS-D) [61], the *Computerized Neuro-psychological Test Battery* (CNTB) [62], *Mindstreams* [63], the *Cognitive Stability Index* (CSI) [64], the *CogState* [15], and the *National Institutes of Health Toolbox-Cognition Battery* (NIHTB-CB) [65]. The cognitive domains assessed across these batteries – including memory, attention, language, executive function, and visuospatial abilities – are similar [10] and align with those included in the CFD. Additionally, most tools (except for the NIHTB-CB) offer alternate forms, as does the CFD, to mitigate learning effects in repeated assessments. In terms of normative sample sizes, the CFD’s normative dataset (n=395) is among the largest, surpassed only by the NIHTB-CB (n=476). The very large samples reported in CANTAB are typically presented as data segmented by cognitive domain [18]. Other batteries such as the CNTB, Mindstreams, and CSI also used reasonably sized samples (>200 participants) [10, 18]. With regards to psychometrics, all reviewed tools report concurrent validity, but construct validity – by means of factor analytic data – was only available for the ANAM, CANTAB, COGDRAS-D, CSI, and NIHTB-CB [10, 17, 18]. Reliability indices, specifically test-retest and internal consistency, were only fully reported for the CNTB, CSI, and NIHTB-CB [10, 17, 18]. Regarding diagnostic performance, while all batteries have been used to identify neurocognitive impairment, the methodological criteria and reporting of criterion validity often do not align with recommended standards [17]. The CSI and CogState stand out for the application of standard diagnostic criteria to compare clinical (MCI or dementia) and cognitively healthy groups, and for providing ROC-based metrics: the CSI demonstrated 80% sensitivity and 87% specificity for dementia but was reported to be particularly difficult for patients with AD [10, 19], whereas CogState reported 78% sensitivity and 90% specificity for MCI [19]. Other tools have shown expected trends of worse performance in AD or dementia versus MCI (e.g., ANAM, CANTAB, COGDRAS-D, CNTB, Mindstreams, NIHTB-CB), but often without reporting sensitivity/specificity analyses [10, 18]. The CFD’s criterion validity was established using DSM-5 criteria to compare participants with NCD and cognitively healthy controls. These analyses confirmed worse performance in the clinical group and demonstrated good sensitivity and specificity (CFD-index: 87%, and 58%, respectively) in NCD discrimination, indicating its potential use as a reliable diagnostic tool beyond screening purposes.

In conclusion, the AACN, the NAN and the European Federation of Psychologists’ Associations (EFPA) [66] have determined that psychological instruments, including computerized tests, require normative and psychometric data to establish validity (construct, concurrent, and criterion validity, including sensitivity and specificity for clinical diagnoses) and reliability (internal consistency, test-retest reliability). All these recommendations were followed in the CFD, with findings supporting that this is a suitable computerized cognitive battery, with adequate psychometric characteristics in a well-defined and representative normative sample. Furthermore, we found that CFD can discriminate NCD, based on DSM-5 criteria, from cognitively healthy controls, proving its appropriateness to be used as a clinical evaluation instrument, extending beyond a brief screening tool, and with potential use as a comprehensive neuropsychological battery. Future studies should address the validity of the CFD and its specific indices for specific neurocognitive diagnosis, enhancing sample sizes to assess predominant impairment in certain cognitive domains.

## Supplementary Materials

The Supplementary data can be found online at: www.aginganddisease.org/EN/10.14336/AD.2025.0412.



## Data Availability

The data that support the findings of this study are available from SCHUHFRIED GmbH but restrictions apply to the availability of these data, which were used under license for the current study, and so are not publicly available. Data are, however, available from the authors upon reasonable request and with permission of SCHUHFRIED GmbH.
