# Supplementary material for: The Cognitive Functions Dementia Battery: A Novel Computerized Tool for Neurocognitive Disorders
Source: Aging Dis. 2025 May 18;17(3):1499–515. doi: 10.14336/AD.2025.0412 (PMC13061557; doi:10.14336/AD.2025.0412)
Supplement: Supplementary file 1 [file AD-17-3-1499-s.pdf]

The Cognitive Functions Dementia Battery: A Novel Computerized Tool for Neurocognitive Disorders

Raquel Lemos, Sofia Areias-Marques, Alexandros Lazaridis, Johanna Zils, Cristina Martins, Sílvia Almeida, Luísa Alves, David Brieber, Albino J. Oliveira-Maia

© 2025. Lemos R et al. Published online at http://www.aginganddisease.org/EN/10.14336/AD.2025.0412

SUPPLEMENTARY ITEMS

Supplementary Table 1. Descriptive Statistics of test main variables for the first (S1) and second (S2) test form

Test	Test forms		Variable	N	mean	sd	min	max	sk	ku
WIWO	S1 (T1)		Verbal fluency – semantic	395	26.11	8.62	9	52	0.62	0.03
	S2 (T2)			362	31.16	9.07	8	71	0.29	0.47
	S1 (T1)		Verbal fluency – lexical	395	15.81	7.06	0	37	0.17	0.05
	S2 (T2)			362	13.89	6.82	0	38	0.52	0.11
AWLT	S1 (T1)		Learning total	395	26.79	6.43	8	43	-0.16	-0.12
	S2 (T2)			362	28.26	7.22	6	43	-0.49	-0.08
	S1 (T1)		Short-term delayed recall	395	7.07	2.30	0	12	-0.37	0.07
	S2 (T2)			362	7.28	2.69	0	12	-0.49	-0.22
	S1 (T1)		Long-term delayed recall	395	6.73	2.50	0	12	-0.35	-0.26
	S2 (T2)			362	6.94	2.68	0	12	-0.33	-0.45
	S1 (T1)		Recognition	395	4.85	1.39	0.6	6.44	-0.62	-0.18
	S2 (T2)			362	4.93	1.38	0.36	6.44	-0.64	-0.31
WAF	A	S1 (T1)	Mean reaction time – intrinsic	395	259.81	44.91	168	531	1.49	4.82
		S1 (T2)		362	256.68	44.45	143	480	1.30	3.48
		S1 (T1)	Mean reaction time – cross modal phasic	395	291.83	57.79	192	540	1.11	1.99
		S1 (T2)		362	289.48	60.81	165	596	1.46	3.81
	G	S1 (T1)	Mean reaction time	395	727.97	201.13	364	1536	0.71	0.56
		S1 (T2)		362	688.38	201.25	280	1479	0.86	0.84
CORSI	S7 (T1)		Immediate block span	395	3.74	1.19	0	7	-0.79	1.86
	S7 (T2)			362	3.92	1.11	0	7	-0.73	2.01
TMT-L	S1 (T1)		Working time Part A	395	19.59	8.04	8.4	83.84	2.42	11.96
	S2 (T2)			362	18.39	7.44	8.43	67.95	2.43	10.61
	S1 (T1)		Working time Part B	395	69.56	57.74	15.06	418.91	2.44	7.59
	S2 (T2)			362	59.03	45.78	12.35	311.6	2.44	7.71
WOBT	S1 (T1)		Object naming	395	22.75	3.77	9	30	-0.61	-0.01
	S2 (T2)			362	23.03	3.78	10	30	-0.59	0.25
VISCO	S1 (T1)		Visuoconstruction ability	395	11.01	9.81	0	32	0.61	-1.02
	S2 (T2)			362	14.28	10.42	0	32	0.18	-1.46

Notes: For each test of the CFD battery, mean, standard deviation (sd), min, max, skewness (sk), and kurtosis (ku) are displayed.
WIWO – Vienna Verbal Fluency Test; AWLT – Auditory Word List Learning Test; WAF - Perception and Attention Functions – Subtest Divided Attention; CORSI - CORSI Block-Tapping Test; TMT-L – Trail Making Test – Langensteinbach Version; WOBT – Vienna Object Naming Test; VISCO – Visuoconstruction Test.
T1 – first test session; T2 – second test session.

Supplementary Table 2. Descriptive Statistics of the CFD indices for the first session (T1) and the re-testing session (T2)

	Index	n	mean	sd	min	max	sk	ku	Percentiles		
									25	50	75
Test session 1	Attention	395	-0.29	1.54	-5.82	3.23	-0.60	0.44	-1.25	-0.10	0.77
	Verbal long-term memory	395	-0.90	2.94	-10.25	5.26	-0.40	-0.12	-2.79	-0.66	1.24
	Expressive language	395	-1.99	1.72	-7.28	2.11	-0.13	-0.27	-3.26	-1.88	-0.80
	Executive functions	395	-0.79	1.68	-8.39	2.23	-1.55	3.17	-1.40	-0.39	0.27
	Perceptual motor functions	395	-0.62	1.02	-1.76	1.57	0.61	-1.02	-1.56	-1.04	0.32
	CFD-Index	395	-3.13	4.51	-18.61	6.43	-0.44	-0.03	-6.01	-2.95	0.44
Test session 2	Attention	362	-0.30	1.56	-5.82	3.23	-0.61	0.45	-1.27	-0.11	0.77
	Verbal long-term memory	362	-0.85	2.98	-10.25	5.26	-0.41	-0.17	-2.80	-0.58	1.33
	Expressive language	362	-1.97	1.73	-7.28	2.11	-0.11	-0.30	-3.23	-1.87	-0.79
	Executive functions	362	-0.80	1.69	-8.39	2.23	-1.59	3.31	-1.38	-0.39	0.24
	Perceptual motor functions	362	-0.62	1.02	-1.76	1.57	0.59	-1.04	-1.56	-0.93	0.32
	CFD-Index	362	-3.12	-4.54	-18.61	6.43	-0.42	-0.07	-6.05	-2.96	0.52

Notes: For each index the CFD battery, mean, standard deviation (sd), min, max, skewness (sk), kurtosis (ku), and 25/50/75 percentiles are displayed.

© 2025. Lemos R et al. Published online at http://www.aginganddisease.org/EN/10.14336/AD.2025.0412

Supplementary Table 3. Structural relations across all tests of the final norm sample (test session 1 – T1).

CFD-T1		AWLT				CORSI	TMT		VISCO	WIWO		WOBT	WAF		
		**LEG**	**KME**	**LME**	**WDI**	**UBS**	**BTA**	**BTB**	**VISCO**	**WOF_S1**	**WOF_S3**	**RBU**	**LMRTR1_i1**	**LMRTR1_i2**	**LMRTR2**
**AWLT**	**KME**	0.81													
	**LME**	0.80	0.90												
	**WDI**	0.68	0.64	0.68											
**CORSI**	**UBS**	0.40	0.38	0.36	0.37										
**TMT**	**BTA**	-0.43	-0.37	-0.37	-0.36	-0.46									
	**BTB**	-0.44	-0.37	-0.36	-0.34	-0.47	0.65								
**VISCO**	**VISCO**	0.40	0.35	0.34	0.38	0.54	-0.47	-0.48							
**WIWO**	**WOF_S1**	0.39	0.39	0.35	0.28	0.36	-0.38	-0.42	0.47						
	**WOF_S3**	0.44	0.38	0.34	0.39	0.44	-0.45	-0.48	0.51	0.59					
**WOBT**	**RBU**	0.47	0.42	0.42	0.45	0.47	-0.50	-0.54	0.55	0.58	0.59				
**WAF**	**LMRTR1_i1**	-0.28	-0.21	-0.20	-0.27	-0.34	0.28	0.31	-0.35	-0.31	-0.34	-0.36			
	**LMRTR1_i2**	-0.29	-0.22	-0.21	-0.27	-0.33	0.30	0.29	-0.33	-0.27	-0.32	-0.32	0.73		
	**LMRTR2**	-0.24	-0.19	-0.19	-0.19	-0.28	0.27	0.25	-0.34	-0.27	-0.26	-0.25	0.56	0.64	
	**LMRTC3**	-0.34	-0.26	-0.25	-0.30	-0.31	0.31	0.25	-0.45	-0.28	-0.31	-0.33	0.38	0.38	0.36

All Pearson correlations depicted are significant at the level of 0.01.

Note: AWLT: Auditory Word List Learning Test; CORSI: CORSI Block-Tapping Test; TMT-L: Trail-Making Test – Langensteinbach Version; VISCO: Visuoconstruction Test; WAF: Perception and Attention Functions; WIWO: Vienna Verbal Fluency Test; WOBT: Vienna Object Naming Test; LEG: Learning total; KME: Short-term delayed recall; LME: Long-term delayed recall; WDI: Recognition; UBS: Immediate block span; BTA: Working time – Part A; BTB: Working time – Part B; VISCO: Visuoconstruction ability; WOF_S1: Verbal fluency – Semantic; WOF_S3: Verbal fluency – Lexical; RBU: Object naming; LMRTRT_i1 & LMRTR1_i2: Alertness – intrinsic (visual) ; LMRTR2: Alertness – cross-modal phasic (visual); LMRTC3: Divided attention – cross-modal (visual/auditory).

Supplementary Table 4. Structural relations across all tests of the final norm sample (test session 2 – T2)

CFD-T2		AWLT				CORSI	TMT		VISCO	WIWO		WOBT	WAF		
		**LEG**	**KME**	**LME**	**WDI**	**UBS**	**BTA**	**BTB**	**VISCO**	**WOF_S2**	**WOF_S4**	**RBU**	**LMRTR1_i1**	**LMRTR1_i2**	**LMRTR2**
**AWLT**	**KME**	0.84													
	**LME**	0.85	0.91												
	**WDI**	0.65	0.66	0.67											
**CORSI**	**UBS**	0.44	0.40	0.37	0.31										
**TMT**	**BTA**	-0.54	-0.45	-0.42	-0.43	-0.49									
	**BTB**	-0.48	-0.40	-0.37	-0.34	-0.50	0.68								
**VISCO**	**VISCO**	0.47	0.42	0.40	0.38	0.58	-0.52	-0.52							
**WIWO**	**WOF_S2**	0.55	0.43	0.44	0.42	0.32	-0.42	-0.41	0.34						
	**WOF_S4**	0.38	0.32	0.30	0.33	0.41	-0.44	-0.41	0.50	0.51					
**WOBT**	**RBU**	0.51	0.46	0.44	0.44	0.43	-0.54	-0.54	0.60	0.41	0.48				
**WAF**	**LMRTR1_i1**	-.299	-0.26	-0.26	-0.20	-0.32	0.41	0.35	-0.40	-0.25	-0.43	-0.34			
	**LMRTR1_i2**	-.298	-0.27	-0.27	-0.20	-0.33	0.38	0.35	-0.35	-0.20	-0.34	-0.33	0.72		
	**LMRTR2**	-.255	-0.21	-0.21	-0.21	-0.29	0.37	0.32	-0.33	-0.14	-0.35	-0.31	0.63	0.70	
	**LMRTC3**	-.406	-0.35	-0.31	-0.33	-0.38	0.45	0.40	-0.50	-0.26	-0.36	-0.36	0.46	0.44	0.42

All Pearson correlations depicted are significant at the level of 0.01.

Note: AWLT: Auditory Word List Learning Test; CORSI: CORSI Block-Tapping Test; TMT-L: Trail-Making Test – Langensteinbach Version; VISCO: Visuoconstruction Test; WAF: Perception and Attention Functions; WIWO: Vienna Verbal Fluency Test; WOBT: Vienna Object Naming Test; LEG: Learning total; KME: Short-term delayed recall; LME: Long-term delayed recall; WDI: Recognition; UBS: Immediate block span; BTA: Working time – Part A; BTB: Working time – Part B; VISCO: Visuoconstruction ability; WOF_S1: Verbal fluency – Semantic; WOF_S3: Verbal fluency – Lexical; RBU: Object naming; LMRTRT_i1 & LMRTR1_i2: Alertness – intrinsic (visual) ; LMRTR2: Alertness – cross-modal phasic (visual); LMRTC3: Divided attention – cross-modal (visual/auditory).

Supplementary Table 5. Coefficients for internal consistency (Cronbach's alpha) and re-test reliability (ICC) for all tests and variables.

Test		Test forms (T1 / T2)	Test variables	Cronbach's α	Re- Test (ICC)
WIWO		S1 / S2	Verbal fluency – semantic	0.59 / 0.56	0.68
		S3 / S4	Verbal fluency – lexical	0.77 / 0.75	0.85
AWLT		S1 / S2	Learning total	0.88 / 0.90	0.85
			Short-term delayed recall	0.56 / 0.69	0.82
			Long-term delayed recall	0.64 / 0.68	0.84
			Recognition	0.64 / 0.65	0.70
WAF	A	S1 / S1	Mean reaction time – intrinsic	0.81 / 0.83	0.73
		S1 / S1	Mean reaction time – cross modal phasic	0.82 / 0.86	0.75
	G	S1 / S1	Mean reaction time	0.87 / 0.88	0.85
CORSI		S7 / S7	Immediate block span	0.82 / 0.83	0.75
TMT-L		S1 / S2	Working time Part A	0.71 / 0.77	0.83
			Working time Part B	0.82 / 0.83	0.86
WOBT		S1 / S2	Object naming	0.80 / 0.76	0.89
VISCO		S3 / S4	Visuoconstruction ability	0.95 / 0.96	0.95

Notes: WIWO – Vienna Verbal Fluency Test; AWLT – Auditory Word List Learning Test; WAF - Perception and Attention Functions – Subtest Divided Attention; CORSI - CORSI Block-Tapping Test; TMT-L – Trail Making Test – Langensteinbach Version; WOBT – Vienna Object Naming Test; VISCO – Visuoconstruction Test.
T1 – Test session 1 (N=395); T2 – Test session 2 (N=362).
ICC – Intraclass correlation coefficient.

© 2025. Lemos R et al. Published online at http://www.aginganddisease.org/EN/10.14336/AD.2025.0412

Supplementary Table 6. Comparison between performances in the first (T1) and the second (T2) sessions

Test	Test forms		Variable	mean T1	mean T2	mean difference	*t*	*p*	Cohen's d
WIWO	S1/S2		Verbal fluency – semantic	26.15	31.16	5.00	-7.59	<0.001*	-0.56
	S3/S4		Verbal fluency – lexical	15.86	13.89	-1.97	3.82	<0.001*	0.28
AWLT	S1/S2		Learning total	26.85	28.26	1.41	-2.77	0.006	-0.21
			Short-term delayed recall	7.12	7.28	0.16	-0.87	0.39	-0.06
			Long-term delayed recall	6.79	6.94	0.16	-0.81	0.42	-0.06
			Recognition	4.86	4.93	0.08	-0.73	0.46	-0.05
WAF	A	S1	Mean reaction time – intrinsic	259.35	256.68	-2.67	0.80	0.42	0.06
		S1	Mean reaction time – cross modal phasic	292.75	289.48	-3.27	0.74	0.46	0.06
	G	S1	Mean reaction time	729.71	688.38	-41.33	2.74	0.006	0.20
CORSI	S7		Immediate block span	3.72	3.92	0.19	-2.27	0.02	-0.17
TMT-L	S1/S2		Working time Part A	19.73	18.39	-1.33	2.29	0.02	0.17
			Working time Part B	69.75	59.03	-10.72	2.75	0.006	0.2
WOBT	S1/S2		Object naming	22.79	23.03	0.25	-0.87	0.38	-0.06
VISCO	S3/S4		Visuoconstruction ability	11.01	14.28	3.27	-4.35	<0.001*	-0.32

Notes: WIWO – Vienna Verbal Fluency Test; AWLT – Auditory Word List Learning Test; WAF - Perception and Attention Functions – Subtest Divided Attention; CORSI - CORSI Block-Tapping Test; TMT-L – Trail Making Test – Langensteinbach Version; WOBT – Vienna Object Naming Test; VISCO – Visuoconstruction Test.
Comparison between T1 and T2 was carried out by paired sample t-tests with Bonferroni alpha correction resulting in statistically significant mean differences if $p < 0.004$

© 2025. Lemos R et al. Published online at http://www.aginganddisease.org/EN/10.14336/AD.2025.0412

Supplementary Table 7. Differences for CFD test main variables between sex, education and age levels.

Test	Variable	*Sex (males vs. females)*					*Education (levels 1-3 vs. 4-5)*					*Age Younger [50-64 years] vs. older elders [≥65 years]*				
		t	*df*	*sig.*[c]	*mean difference*	*Cohens'd*	*t*	*df*	*sig.*[c]	*mean difference*	*Cohens'd*	*t*	*df*	*sig.*[c]	*mean difference*	*Cohen's d*
WIWO	Verbal fluency – semantic	1.56	393	0.12	1.36	0.16	-8.06	393	<0.001*	-6.62	-0.83	2.46	393	0.014	2.12	0.25
	Verbal fluency – lexical	1.37	393	0.17	0.98	0.14	-10.78	393	<0.001*	-6.87	-1.11	2.06	393	0.040	1.46	0.21
AWLT	Learning total	-2.47	393	0.01	-1.59	-0.25	-5.60	393	<0.001*	-3.56	-0.58	6.78	393	<0.001*	4.15	0.68
	Short-term delayed recall	-0.95	393	0.34	-0.22	-0.10	-3.01	393	0.003	-0.70	-0.31	7.35[a]	376.73	<0.001*	1.60	0.74
	Long-term delayed recall	-1.80	393	0.07	-0.46	-0.18	-2.51	393	0.012	-0.64	-0.26	7.26[a]	371.98	<0.001*	1.72	0.73
	Recognition	-0.94	393	0.35	-0.13	-0.10	-5.00[a]	363.20	<0.001*	-0.68	-0.50	5.50[a]	382.46	<0.001*	0.74	0.55
WAF A	Mean reaction time – intrinsic[b]	-1.48	393	0.14	-6.71	-0.15	6.41[a]	389.31	<0.001*	26.66	0.62	-0.04	393	0.967	-0.19	0.00
	Mean reaction time – cross modal phasic[b]	-3.22[a]	392.97	0.001*	-18.27	-0.32	3.86	393	<0.001*	22.52	0.40	-1.42	393	0.158	-8.22	-0.14
WAF G	Mean reaction time[b]	-3.72	393	<0.001*	-74.36	-0.38	7.02[a]	373.81	<0.001*	132.46	0.70	-3.48	393	0.001*	-69.46	-0.35
CORSI	Immediate block span	1.89	393	0.06	0.23	0.19	-6.07[a]	377.97	<0.001*	-0.68	-0.60	3.90	393	<0.001*	0.46	0.39
TMT-L	Working time Part A[b]	1.12	393	0.26	0.91	0.11	6.97[a]	392.96	<0.001*	5.08	0.66	-5.69[a]	325.96	<0.001*	-4.43	-0.57
	Working time Part B[b]	-1.06	393	0.29	-6.17	-0.11	7.25[a]	390.73	<0.001*	37.28	0.68	-5.45[a]	307.52	<0.001*	-30.62	-0.55
WOBT	Object naming	0.81	393	0.42	0.31	0.08	-9.64[a]	371.67	<0.001*	-3.27	-0.96	2.48	393	0.014	0.93	0.25
VISCO	Visuoconstruction abilit	4.32[a]	359.6	<0.001*	4.23	0.44	-9.17[a]	284.03	<0.001*	-8.70	-0.98	3.59[a]	384.74	<0.001*	3.49	0.36

Notes: WIWO – Vienna Verbal Fluency Test; AWLT – Auditory Word List Learning Test; WAF - Perception and Attention Functions – Subtest Divided Attention; CORSI - CORSI Block-Tapping Test; TMT-L – Trail Making Test – Langensteinbach Version; WOBT – Vienna Object Naming Test; VISCO – Visuoconstruction Test.
Education levels: 1 (≤ 4 years); 2 (5 – 6 years); 3 (7 – 9 years); 4 (10 – 12 years); and 5 (≥ 13 years).
Positive mean difference indicates higher scores for male participants, and for participants with education level 1-3.
[a] Violation of homogeneity assumption -> Welch-test. [b] Higher scores indicate lower ability. [c] The alpha level was Bonferroni corrected (α = 0.0025), significant p-values are marked with an *.

© 2025. Lemos R et al. Published online at http://www.aginganddisease.org/EN/10.14336/AD.2025.0412

Supplementary Table 8. CFD indices differences between Mild Cognitive disorder (MiCD/MCI), Major Cognitive disorder (MaCD/Dementia), and healthy participants

	MiCD/MCI (n=45)	MaCD/Dementia (n=26)	Healthy group (n=196)	*F*	*sig.*[a]	Multiple comparisons
**CFD-Index**	-7.35 (5.62)	-13.46 (5.66)	-3.31 (4.82)	52.42	<0.001*	MCI<HG: p<0.001*<br>Dementia<HG: p<0.001*<br>MCI>Dementia: p<0.001*
**Attention**	-1.80 (2.15)	-3.84 (3.08)	-0.28 (1.49)	50.72	<0.001*	MCI<HG: p<0.001*<br>Dementia<HG: p<0.001*<br>MCI>Dementia: p<0.001*
**Verbal long-term memory**	-4.29 (3.52)	-7.89 (2.85)	-1.15 (3.09)	63.11	<0.001*	MCI<HG: p<0.001*<br>Dementia<HG: p<0.001*<br>MCI>Dementia: p<0.001*
**Expressive language**	-2.91 (1.70)	-4.84 (1.61)	-1.86 (1.75)	37.04	<0.001*	MCI<HG: p=0.001*<br>Dementia<HG: p<0.001*<br>MCI>Dementia: p<0.001*
**Executive functions**	-1.63 (2.24)	-3.28 (2.96)	-0.94 (1.92)	11.66	<0.001*	MCI<HG: p=0.14<br>Dementia<HG: p<0.001*<br>MCI>Dementia: p=0.01
**Perceptual motor functions**	-0.88 (1.01)	-1.13 (0.87)	-0.64 (0.98)	3.09	0.05	N/A

Notes: MiCD – Mild Cognitive Disorder; MCI – Mild Cognitive Impairment; MaCD – Major Cognitive Disorder
Data are expressed as mean (SD). Comparisons between the two groups were carried out by one-way ANOVA with post-hoc multiple comparisons with Bonferroni corrections.
[a] The alpha level was Bonferroni corrected ($\alpha = 0.008$), significant p-values are marked with an *.

© 2025. Lemos R et al. Published online at http://www.aginganddisease.org/EN/10.14336/AD.2025.0412

Supplementary Table 9. Diagnostic classification accuracy of the CFD indices in discriminating Mild Cognitive disorder (MiCD/MCI), Major Cognitive disorder (MaCD/Dementia), from the cognitively healthy group.

	MiCD/MCI					MaCD/Dementia				
	AUC	95% CI	*sig.*	Sensitivity	Specificity	AUC	95% CI	*sig.*	Sensitivity	Specificity
**CFD-Index**	0.72	[0.66, 0.78]	<0.0001	84.44	55.61	0.91	[0.87, 0.94]	<0.0001	84.62	82.14
**Attention**	0.72	[0.65, 0.77]	<0.0001	75.00	66.33	0.85	[0.80, 0.90]	<0.0001	69.23	94.90
**Verbal long-term memory**	0.74	[0.68, 0.80]	<0.0001	57.78	85.71	0.94	[0.90, 0.96]	<0.0001	76.92	96.43
**Expressive language**	0.68	[0.62, 0.74]	<0.0001	75.00	59.18	0.90	[0.85, 0.93]	<0.0001	96.15	73.47
**Executive functions**	0.63	[0.56, 0.69]	0.004	67.44	59.69	0.76	[0.70, 0.81]	<0.0001	66.67	84.18
**Perceptual motor functions**	*0.59*	*[0.53, 0.66]*	*0.07*	*50.00*	*68.88*	0.67	[0.61, 0.74]	0.008	60.00	75.00

Notes: MiCD – Mild Cognitive Disorder; MCI – Mild Cognitive Impairment; MaCD – Major Cognitive Disorder
AUC – area under the operating characteristics curve; CI – confidence interval. Sensitivity and Specificity values are expressed in percentage.

© 2025. Lemos R et al. Published online at http://www.aginganddisease.org/EN/10.14336/AD.2025.0412
